# Strain Effect on Thermoelectric Performance of InSe Monolayer

**DOI:** 10.1186/s11671-019-3113-9

**Published:** 2019-08-19

**Authors:** Qian Wang, Lihong Han, Liyuan Wu, Tao Zhang, Shanjun Li, Pengfei Lu

**Affiliations:** 1 State Key Laboratory of Information Photonics and Optical Communications, Beijing University of Posts and Telecommunications, Beijing, 100876 China; 2 College of Electrical Engineering and Information Technology, Sichuan University, Chengdu, 610065 China

**Keywords:** Two-dimensional materials, Thermoelectric, Strain engineering

## Abstract

Strain engineering is a practical method to tune and improve the physical characteristics and properties of two-dimensional materials, due to their large stretchability. Tensile strain dependence of electronic, phonon, and thermoelectric properties of InSe monolayer are systematically studied. We demonstrate that the lattice thermal conductivity can be effectively modulated by applying tensile strain. Tensile strain can enhance anharmonic phonon scattering, giving rise to the enhanced phonon scattering rate, reduced phonon group velocity and heat capacity, and therefore lattice thermal conductivity decreases from 25.9 to 13.1 W/mK when the strain of 6% is applied. The enhanced figure of merit indicates that tensile strain is an effective way to improve the thermoelectric performance of InSe monolayer.

**Electronic supplementary material:**

The online version of this article (10.1186/s11671-019-3113-9) contains supplementary material, which is available to authorized users.

## Introduction

Two-dimensional (2D) semiconductor materials have been drawing the attention of researcher to explore their fascinating properties and useful application since the discovery of graphene. Especially, the family of two-dimensional metal-chalcogenide has been found to show great potential in nanoelectronics and nanophotonics due to their extraordinary electronic, optical, and mechanical properties [1–4]. Recently, indium selenide (InSe), a III-VI group layered metal-chalcogenide compound, is of great interest both experimentally and theoretically. The atomic layer of InSe has been reported to successfully synthesized via physical [5–10] and chemical methods [11–14], and the applications of InSe nanosheet on sensors [15], optoelectronics, and photodetectors have been explored. Srinivasa et al. reported the fabrication of few-layer InSe photodetectors with high responsivity and a broad spectral detection from the visible to near-infrared region [6]. Bandurin et al. found a high-quality two-dimensional electron gas in few-layer InSe with the carrier mobilities of 10^3^ and 10^4^ cm^2^/Vs at room and liquid-helium temperatures [16]. Wei et al. discovered back-gated multilayer InSe FETs exhibit ultrahigh carrier mobility up to 1055 cm^2^/Vs at room temperatures due to suppressed carrier scattering from the dielectric substrate [5].

2D InSe has a rather unusual band structure, which is the combination of a flat band at the top of the valence band and parabolic band at the bottom of the conduction band, thus exhibiting high thermoelectric characteristics [17]. Particularly, thermoelectric performance can be described by the non-dimensionalized figure of merit, *ZT*, defined as *ZT = S*^*2*^*Tσ/*(*Κ*_e_ + *Κ*_l_), where *S* is the Seebeck efficient, *T* is the absolute temperature, *σ* is the electrical conductivity, and *Κ*_e_ and the *Κ*_l_ are the thermal conductivity with the contributions from electronic carriers and lattice, respectively. The lattice thermal conductivity *K*_*l*_ relevant to phonon transport property plays an important role to determine the thermoelectric performance. The previous reported *K*_l_ of InSe monolayer is much lower than that of graphene, while it was 10 times as much as that of SnSe sheet [18, 19].

The high level of electron mobility and low thermal conductivity is beneficial to the thermoelectric performance. Besides, monolayer InSe exhibits superior mechanical flexibility, and the electronic properties can be continuously modulated by moderate strain in a wide range [20–22]. It has been demonstrated that the thermoelectric power factor of monolayer InSe can be significantly enhanced through band convergence under a compressive strain [23]. For thermoelectric materials, tensile strain can also induce a variation of band structure and thermal transport properties. However, the dependence of thermal transport properties on the strain is unpredictable, closely related to the particular material and crystal structure. In this paper, the present work is performed on the biaxial tensile strain effect for the thermoelectric performance of InSe monolayer by first-principles calculations, including electronic and phonon transport properties. Due to the increased anharmonic scattering, the positive effect of tensile strain on the thermoelectric performance of InSe monolayer is determined.

## Methodology

The calculation of the structural and electronic properties for InSe monolayer are performed based on density functional theory (DFT) as implemented in the Vienna ab initio simulation package (VASP) [24–26]. We chose the projector-augmented wave method with the local density approximation (LDA) [27–29] for the exchange-correlation functional. And 12 Å vacuum along the *z*-axis is used to avoid the interaction between periodic images of slabs. The 21 × 21 × 1 and 31 × 31 × 1 Monkhorst-Pack k-meshes were used during structural relaxation and electronic structure calculations for the unit cell. The energy cutoff of the plane wave basis was set to be 500 eV. The convergence criterion for a total energy was set as 10^−4^ eV, and all the atomic positions and lattice structures were fully relaxed with a force tolerance of 10^−3^ eV/Å.

The thermoelectric transport properties can be obtained within the constant relaxation time approximation by the Boltzmann theory as implemented in BoltzTraP program [30, 31]. Within this approximation, the electronic transport coefficients can be given by

1 $$ {S}_{\alpha \beta}\left(T,\mu \right)=\kern0.3em \frac{1}{\mathrm{e}T\Omega {\sigma}_{\alpha \beta}\left(T,\mu \right)}\int {\sum}_{\alpha \beta}\left(\varepsilon \right)\left(\varepsilon -\mu \right)\left[-\frac{\partial {f}_{\mu}\left(T,\varepsilon \right)}{\partial \varepsilon}\right] d\varepsilon $$

2 $$ {\sigma}_{\alpha \beta}\left(T,\mu \right)\kern0.3em =\kern0.3em \frac{1}{\Omega}{\int \sum}_{\alpha \beta}\left(\varepsilon \right)\left[-\frac{\partial {f}_{\mu}\left(T,\varepsilon \right)}{\partial \varepsilon}\right] d\varepsilon $$

where Ω is the volume of the unit cell, *f*_*μ*_ is the Fermi-Dirac distribution function, and *α* and *β* are tensor indices. The transport distribution function ∑_*αβ*_(*ε*) is given by

3 $$ {\sum}_{\alpha \beta}\left(\varepsilon \right)\kern0.3em =\kern0.3em \frac{e^2}{N_0}\sum \limits_{i,\mathrm{q}}\tau {v}_a\left(i,\mathrm{q}\right){v}_{\beta}\left(i,\mathrm{q}\right)\frac{\delta \left(\varepsilon -{\varepsilon}_{i,\mathrm{q}}\right)}{d\varepsilon} $$

where *N*_0_ indicates the number of *q* points sampled, *i* is the band index, *v* is the group velocity of carriers, and *τ* is the relaxation time.

The ShengBTE package [32] is employed to solve the phonon Boltzmann transport equation and determine the lattice thermal and other relevant parameters. A 5 × 5 × 1 supercell is used to calculate the harmonic interatomic force constants by using density-functional perturbation theory (DFPT) calculation [33]. And the finite-difference method is used to calculate anharmonic interatomic force constants with a 4 × 4 × 1 supercell [34]. Phonon spectrum was calculated by using the Phonopy program [35].

## Result and Discussion

**Fig. 1:**
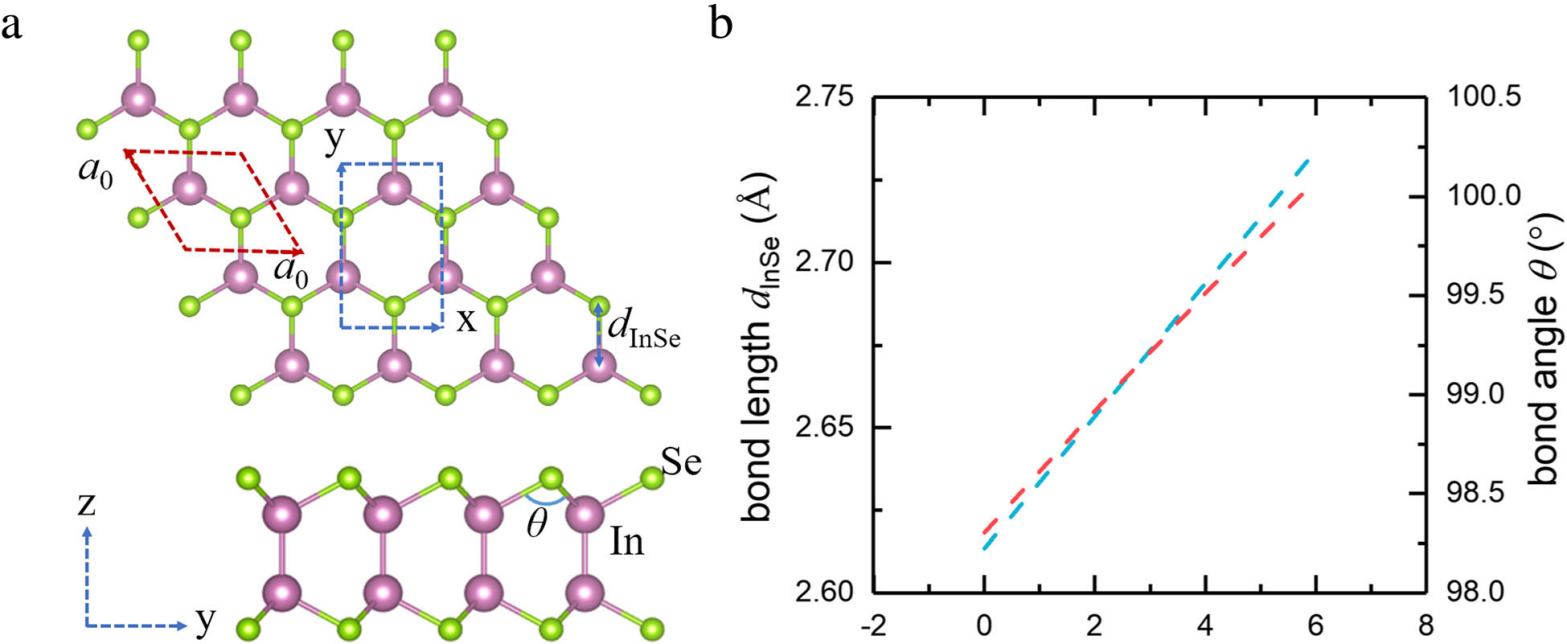
**a** Top view and side view of monolayer InSe. Pink and green balls represent In and Se atoms, respectively. **b** The variation of bond length and bond angle with the increase of biaxial tensile strain. The basic *a*_0_ × *a*_0_ unit cell and *x* × *y* supercell of InSe monolayer are denoted with red and blue dashed lines, respectively

Monolayer InSe is a quadruple atomic sheet with Se-In-In-Se covalently bonding in one layer. From a top view, the monolayer exhibits a honeycomb lattice, and every Se atom is bonded with other three In atoms, as shown in Fig. 1a. On the basis of the minimization of the total energy, the lattice parameters of this crystal are calculated to be *a*_0_ = 3.95 Å. In this paper, we employ the biaxial strain on monolayer InSe maintaining the crystal symmetry by changing its lattices as *δ* = (*a*−*a*_0_)/*a*_0_ × 100%, where *a* and *a*_0_ are the lattice constant of monolayer InSe with strain and without strain, respectively. When the biaxial tensile strain is imposed on the monolayer InSe, the bond length *d*_InSe_ monotonically increase with the increase of strain, and this lead to the increasing bond angle of In-Se-In (see Fig. 1b).

**Fig. 2:**
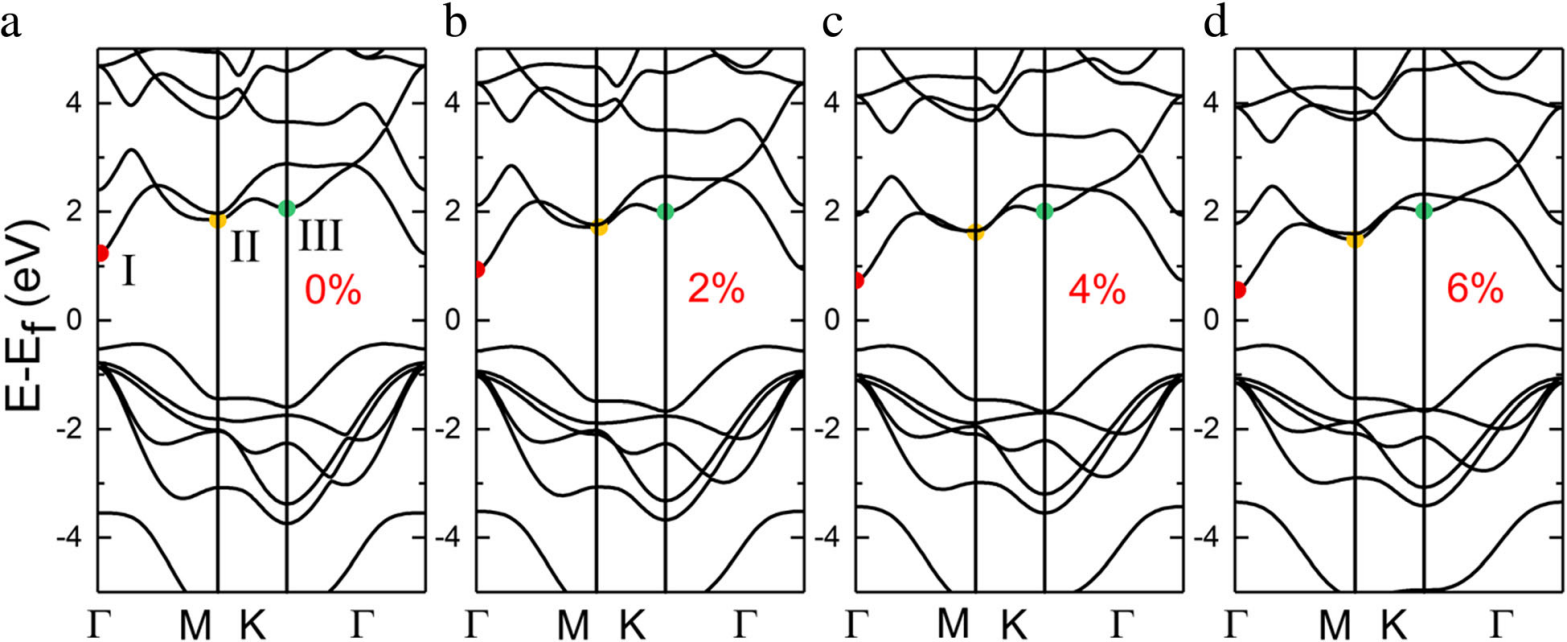
Band structure of InSe monolayer under different strain condition

InSe monolayer exhibits an indirect semiconductor with the bandgap of 1.67 eV, where the conduction band minimum (CBM) exists on the Г point, and valence band maximum (VBM) sites on between Г and K point, as shown in Fig. 2a. The valence band of InSe monolayer exhibits a Mexican hat dispersion, which can be also found in many two-dimensional materials [36–39]. The band structure modification in response to tensile strain was investigated in Fig. 2, and the three conduction band extrema are denoted by the symbols I, II, and III respectively. Under a tensile strain, the lowest-energy conduction band is sensitive to strain and shifts downwards, while valence band almost remain constant, giving rise to the reduction of the bandgap. Without strain, there are minute differences between the second and third conduction band minimum, and the band valleys are tending to convergence. However, with the increase of tensile strain, the energy difference gradually increases. We also compared the bandgaps under different strains with related theoretical and experimental results as detailed in Additional file 1: Table S2.

### Effect of Tensile Strain on Thermoelectric Transport Coefficients

**Fig. 3:**
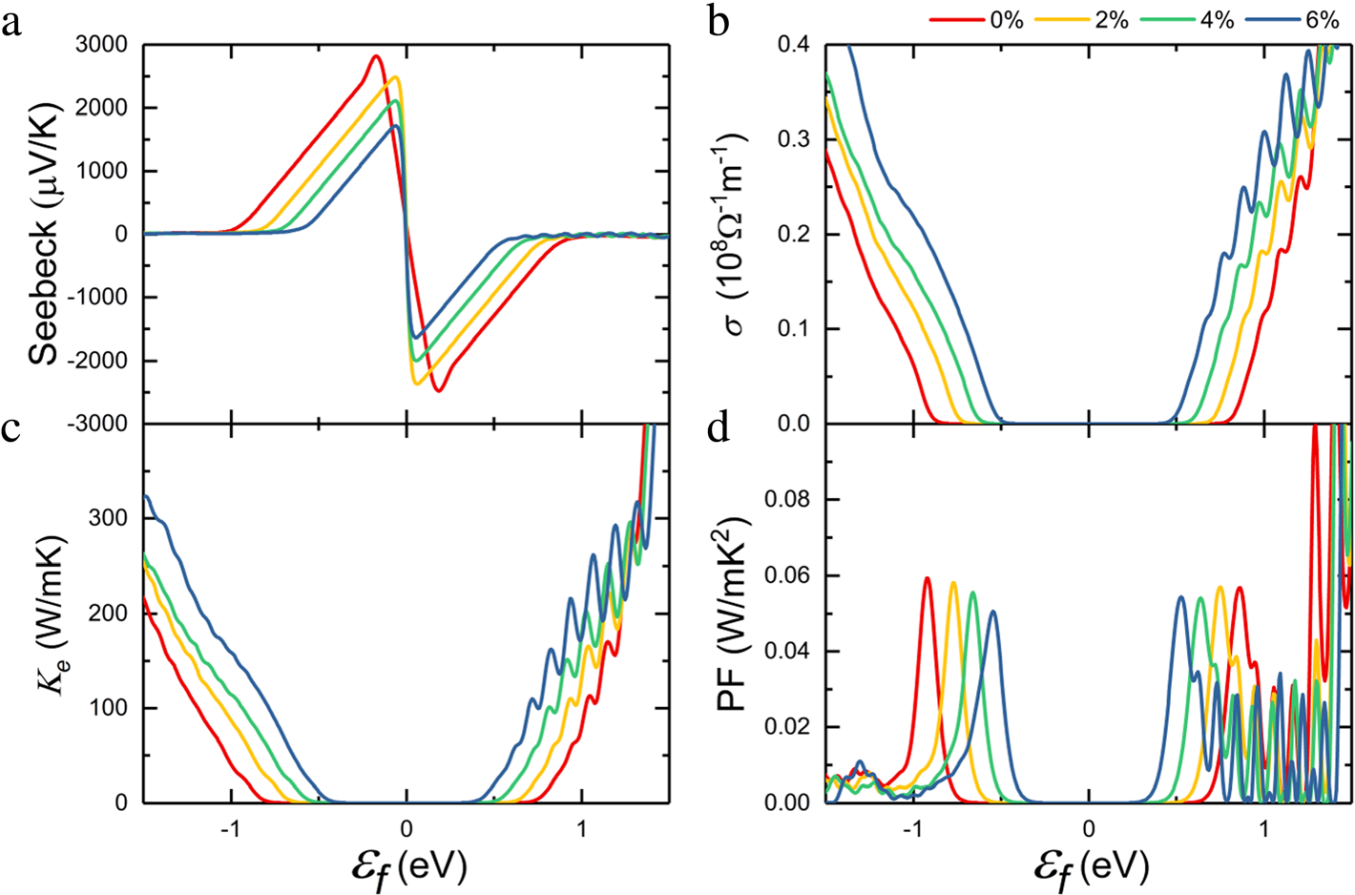
**a** Seebeck coefficient, **b** electrical conductivity, **c** electronic thermal conductivity, **d** power factor of the monolayer InSe as a function of chemical potential at 300 K when the different biaxial strain is applied

On the basis of the calculated electronic structure, we perform thermoelectric transport coefficient calculations by the semi-classical Boltzmann theory. With respect to scattering time *τ*, Seebeck coefficient *S*, and electrical conductivity *σ* can be calculated. Figure 3a shows the calculated Seebeck coefficient as a function of the Fermi level. For simplicity, the band structure is often assumed to remain unchanged from doping at finite temperatures [40, 41], and doping effect on thermoelectric transport coefficient can be obtained by the variation of the position of Fermi level. A negative *ε*_f_ indicates p-type doping by moving the Fermi level into the valence band, and the positive Seebeck coefficient can be obtained. Similarly, a positive *ε*_f_ gave a negative Seebeck coefficient. We can find the obtained result without strain is very close to the previous report [17], and the maximum of Seebeck coefficient decreases with increasing tensile strain, which is related to the change of the bandgap [42].

To calculate the electrical conductivity *σ*, relaxation time *τ* is required because the output is *σ*/*τ* in BoltzTraP code. Herein, *τ* is determined by

4 $$ \mu \kern0.3em =\kern0.3em e\tau /m\ast $$

where *μ* is carrier mobility and m* is the effective mass. In the deformation potential theory, the carrier mobility in 2D materials can be calculated by [43, 44]

5 $$ \mu \kern0.3em =\kern0.3em \frac{e{\mathrm{\hslash}}^3C}{k_B{Tm}^{\ast }{m}_{\mathrm{d}}{E_1}^2} $$

Here, *e* is the electron charge, ℏ is the Planck constant, and *k*_B_ is Boltzmann constant. *C* represents the elastic modulus and can be calculated by *C* =  (*∂*^2^*E*/*∂δ*^2^)/*S*_0_, where *E*, *δ*, and *S*_0_ are the total energy, the applied strain, and the area at equilibrium for the 2D system, respectively. *E*_1_ is deformation potential constant shown as *E*_1_ =  *ΔE*_edge_/*Δδ*, where *ΔE*_edge_ is the energy change of band edges. *m*_d_ is average effective mass derived from ${m}_d=\sqrt{m_x^{\ast }{m}_y^{\ast }}$. In order to calculate the mobility, a rectangular *x* × *y* supercell is adopted as shown in Fig. 1a. The obtained value of *C* along *x* (*y*) direction is 60.43 N/m (53.68 N/m), which is obtained by fitting the curve of energy-strain relationship, as shown in Additional file 1: Figure S1. The calculated deformation potential *E*_1_ is 6.13 eV (6.14 eV) for electron along *x* (*y*) direction, and 3.45 eV (3.33 eV) for hole along *x* (*y*) direction. The calculated results of effective mass, carrier mobility, and relaxation time for monolayer InSe under different strain are summarized in Table 1. We can find that little difference along with different directions, and the carrier effective mass and mobility is general isotropic. Therefore, we use the average value of *x* and *y* directions to evaluate the thermoelectric performance later. The hole effective masses are enhanced by the applied strain while the effective masses for electron remains almost unchanged. With the calculated relaxation time, the electrical conductivity can be obtained at a given chemical potential in Fig. 3b. It can be seen that electrical conductivity *σ* increase with increasing the tensile strain in a heavy p-type doped system due to the enhancement of hole mobility, whereas *σ* remains relatively low at low doping level. Moreover, the trend of electronic thermal conductivity keeps with the electrical conductivity through the Wiedemann-Franz law: *K*_e_ = *LσT* in Fig. 3c, where *L* is the Lorenz number. Power factor can be obtained by PF = *S*^2^*σ* /*τ*, which determines how much electricity can be generated. Considering the comprehensive trend of Seebeck coefficient and electrical conductivity, tensile strain slightly reduces the power factor, as observed in Fig. 3d.

**Table 1:** Calculated electron effective mass m^*^ (*m*_0_), carrier mobility *μ* (cm^2^V^−1^s^−1^), and relaxation time *τ* (fs) of the electron (*e*) and hole (*h*) along the *x* and *y* directions in monolayer InSe at 300 K under different tensile strain. Some previous calculation and experimental results are also listed for comparison

%		*m* ^*^_x_	*m* ^*^_y_	*μ*_*x*_	*μ*_*y*_	*μ*_exp_	*τ*_*x*_	*τ*_*y*_
0	*e*	0.16	0.16	1336	1184	10^3^ [16]	121.70	107.86
		0.19 [60]	0.18 [60]	801.09 [61]	689.20 [61]			
	*h*	2.00	2.00	27	26	40 [62]	30.76	29.39
2	*e*	0.16	0.16	1336	1184	–	121.70	107.86
	*h*	1.80	1.80	33	32	–	34.17	32.65
4	*e*	0.17	0.17	1183	1049	–	114.50	101.53
	*h*	1.69	1.69	38	36	–	36.39	34.78
6	*e*	0.17	0.17	1183	1049	–	114.50	101.53
	*h*	1.40	1.40	55	53	–	43.93	41.98

### Effect of Tensile Strain on *Κ*_l_

In metals, electrons are responsible for heat carriers, while in semiconductors and dielectric solids where the doping and temperature are not very high, lattice vibrations will be the main reason for energy transport [45]. Lattice thermal conductivity is a very important parameter for thermoelectric application. From the theoretical point of view and as a simple approximation, the lattice thermal conductivity *Κ*_l_ can be expressed as follows [46–48]:

6 $$ {K}_{\mathrm{l}}=\frac{1}{V}\sum \limits_{\uplambda}{C}_{\uplambda}{v}_{\uplambda}^2{\tau}_{\uplambda}\kern0.4em $$

where *C*_λ_, *v*_λ_, and *V* are specific heat contribution, phonon group velocity, and crystal volume, respectively. *τ*_λ_ is the relaxation time of mode λ, which can be estimated using the Matthiessen rule [49]:

7 $$ \frac{1}{\tau_{\uplambda}}=\frac{1}{\tau_{\uplambda}^{3\mathrm{ph}}}\kern0.4em +\kern0.5em \frac{1}{\tau_{\uplambda}^b}\kern0.5em +\kern0.4em \frac{1}{\tau_{\uplambda}^{\mathrm{iso}}} $$

where $\frac{1}{\tau_{\uplambda}^b}$is the boundary scattering rate, $\frac{1}{\tau_{\uplambda}^{\mathrm{iso}}}$is the isotropic impurity scattering rate, and $\kern0.1em \frac{1}{\tau_{\uplambda}^{3\mathrm{ph}}}$ is the three-phonon scattering rate.

**Fig. 4:**
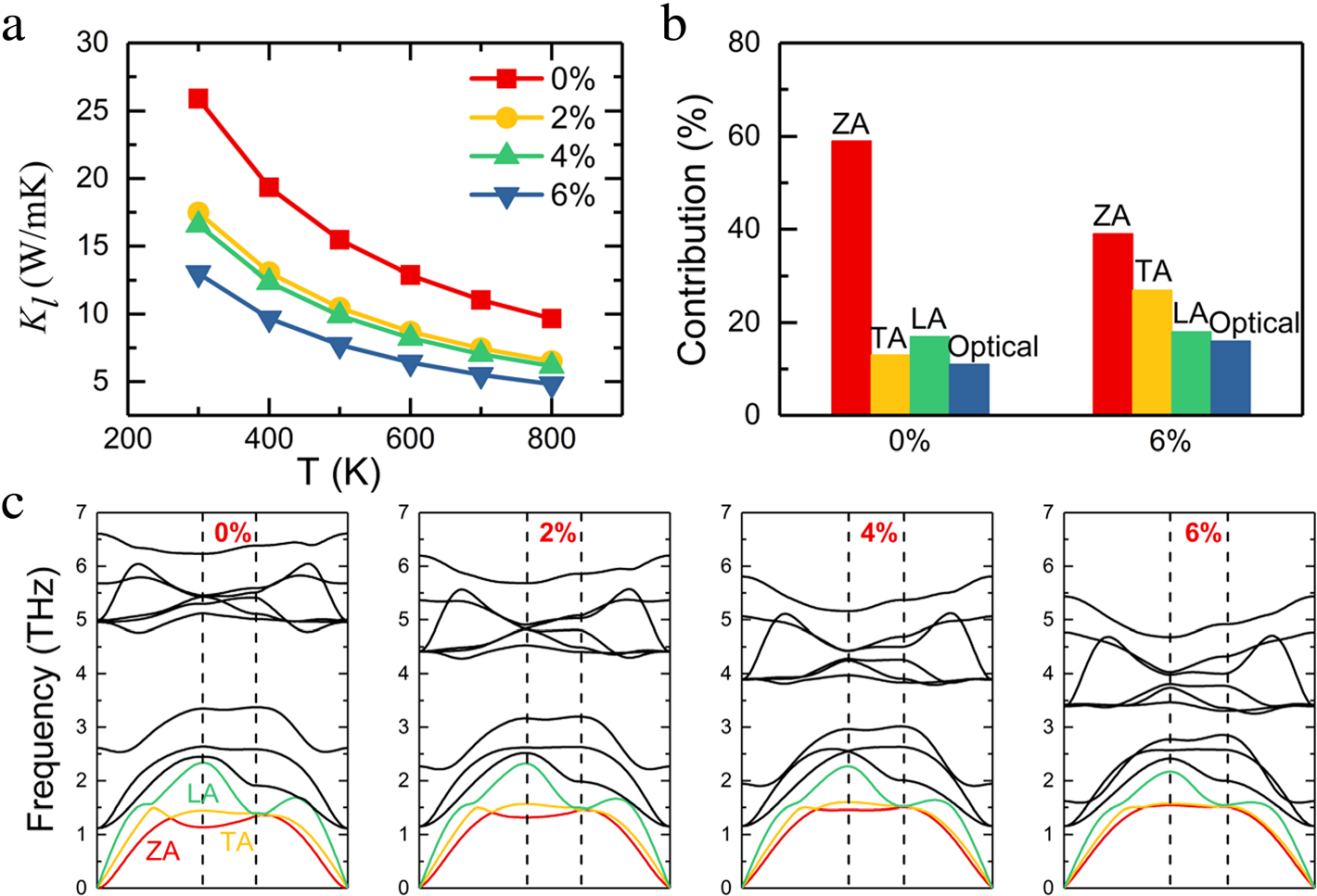
**a** Calculated biaxial strain effects on lattice thermal conductivity at different temperatures. **b** Contribution of the ZA, TA, LA, and all optical branches towards the lattice thermal conductivity for unstrained and 6% strained systems. **c** The phonon dispersion curves of the monolayer InSe for different strains

Figure 4a presents *Κ*_l_ variation of monolayer InSe with temperature under different strain. The lattice thermal conductivity in the strain-free case is 25.9 W/mK at room temperature, which is comparable with the previous report [19]. When the applied strain is increased to 6%, the lattice thermal conductivity decreased to 13.1 W/mK, which confirms that strain engineering is a very efficient method to modify the lattice thermal conductivity. We plot the corresponding phonon dispersion curve of InSe monolayer for different strains in Fig. 4c, to determine the origin of the reduction on lattice thermal conductivity. It contains 12 phonon modes as monolayer InSe has a four-atom unit cell. There is no negative frequency in phonon spectra, confirming that InSe monolayer is thermally stable. Three branches starting from 0 in the low energy region of the phonon dispersion curve are *z*-axis acoustic (ZA), longitudinal acoustic (LA), and transverse acoustic (TA), respectively, and the others are optical modes. With the increase of tensile strain, the quadratic nature of the ZA mode changes into almost a straight line in the low-energy region. The downward trend in the frequency of optical modes can be observed under tensile strain, because tensile strain weakens the bonds and then leads to lower frequencies. We also discuss the contribution of each phonon branch towards *Κ*_l_ for the unstrained and 6% strain monolayer InSe in Fig. 4b. For the strain-free condition, the ZA mode contributes significantly to carrying heat, and when 6% tensile strain is applied to monolayer InSe, the relative contribution of ZA mode is decreased from 58 to 38%. As the tensile strain increases, ZA mode becomes harder, leading to a decreased contribution to *Κ*_*l*_.

**Fig. 5:**
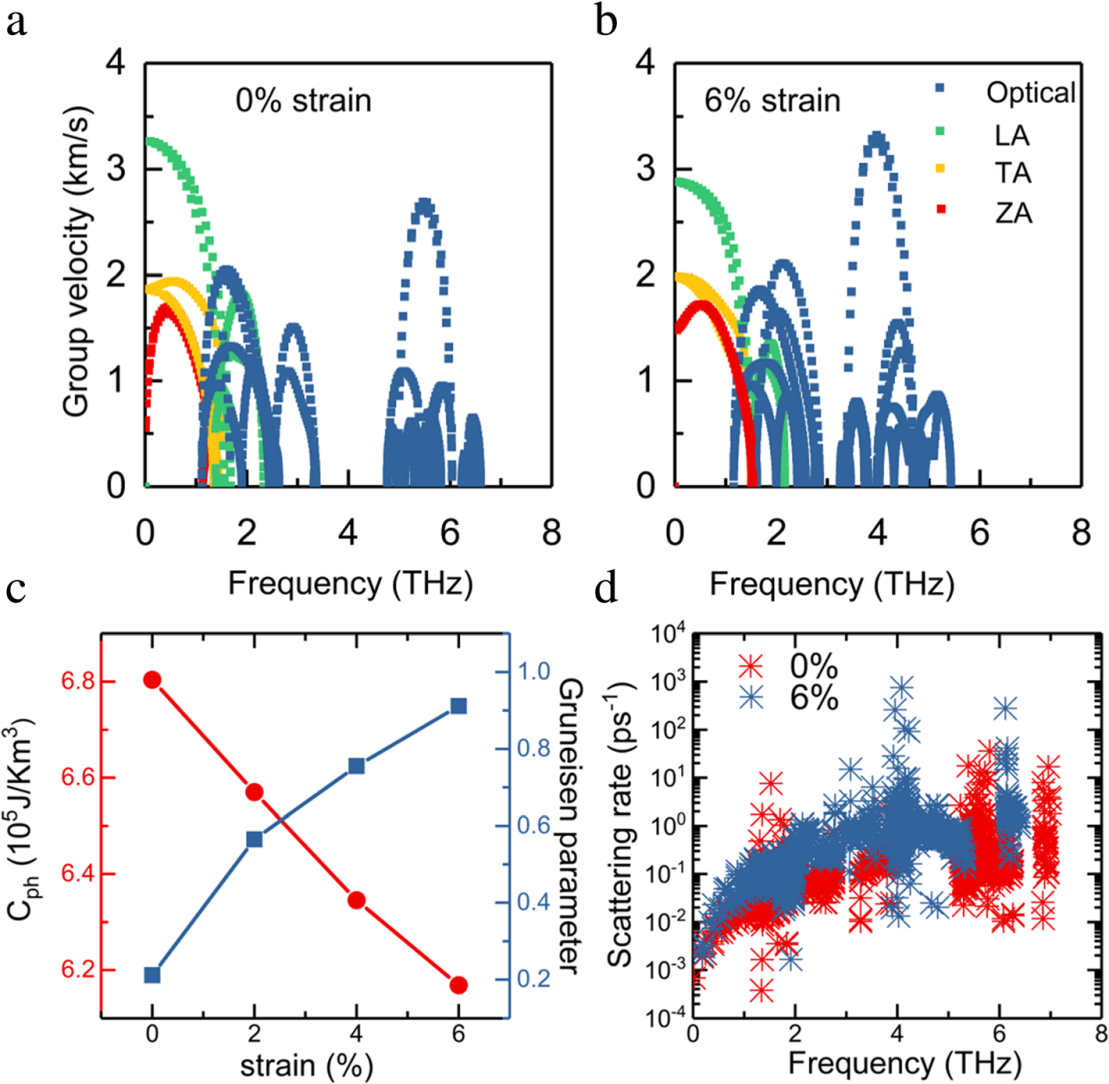
The contribution of ZA, TA, LA, and optical modes to the group velocity of monolayer InSe for (**a**) unstrained and (**b**) 6% strained systems. **c** Phonon heat capacity (*C*_ph_) and Gruneisen parameter as a function of strain at 300 K. **d** Phonon scattering rate of unstrained and 6% strained monolayer InSe as a function of frequency.

Next, a detailed analysis of phonon group velocity variation induced by tensile strain is presented to understand the phonon transport properties. For in-plane acoustic modes, phonon group velocities are decreased at the strain of 6%, as shown in Fig. 5a, b. Combined with the enhanced contribution of LA and TA, decreased phonon group velocity plays a vital role in the reduction of *Κ*_l_. The change of phonon group velocities originates from strain-induced structure variation: when tensile strain is turned on, the bond distance increase and bonding strength decrease, leading to the lower phonon frequency and group velocity. Considering that three acoustic phonon branches contribute mostly to *Κ*_l_, the increased phonon group velocities of optical branches have limited effect.

The three-phonon scattering rate of monolayer InSe without and with 6% strain as a function of frequency is depicted in Fig. 5d. It can be observed that three-phonon scattering rate of 6% strained monolayer InSe in the lower frequency region is significantly larger than that of the unstrained case, which indicates that the increase of strain gives rise to stronger three-phonon scattering. The enhanced three-phonon scattering is mostly responsible for the reduced lattice thermal conductivity, which is also consistent with previous conclusion [19]. A similar trend of phonon scattering rate with the increased tensile strain has been observed in ZrS_2_ and 2H MoTe_2_ monolayer [50, 51]. We also analyzed the effect of biaxial tensile strain on phonon heat capacity (*C*_*ph*_), as presented in Fig. 5c. With the increase of tensile strain, the phonon heat capacity of InSe monolayer is monotonously decreased. For the 6% strained system, the phonon heat capacity is reduced to 6.2 × 10^5^ J/Km^3^. Because of the linearization and stiffening of the ZA mode, the phonon density of states is decreased, leading to the reduced phonon heat capacity. The Gruneisen parameters provide information about the anharmonicity of a system and can be obtained from the anharmonic interatomic force constants (IFCs) [32, 52]. Figure 5c displays the calculated Gruneisen parameters under different strains. The increased Gruneisen parameter induced by the tensile strain means stronger anharmonicity, leading to lower thermal conductivity [18].

**Fig. 6:**
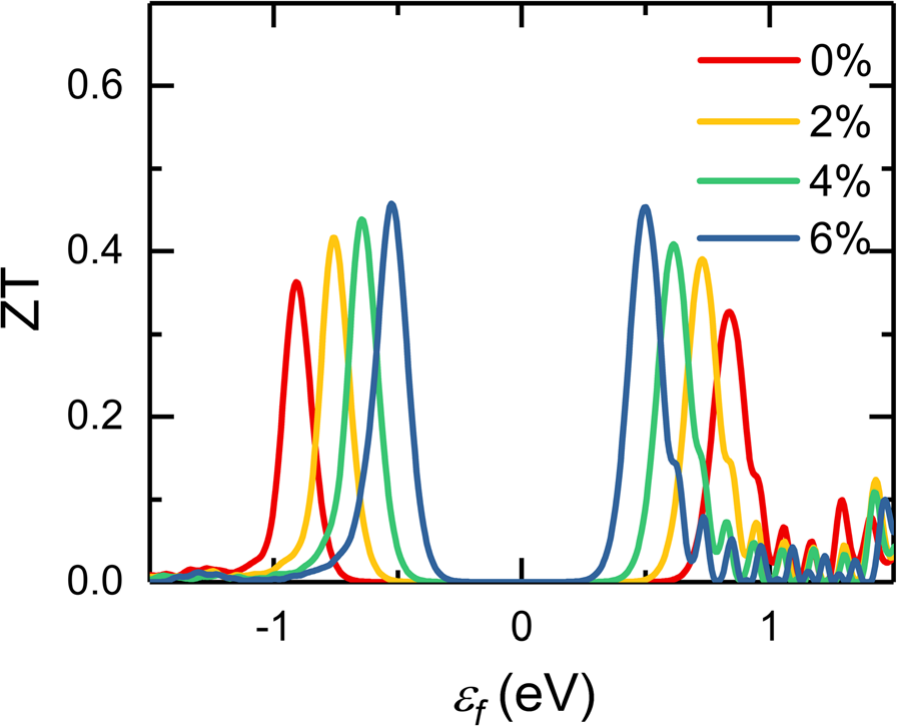
Calculated figure of merit of monolayer InSe as a function of chemical potential under different strain

With all the thermoelectric transport properties available, the figure of merit, ZT, can be obtained. The applied tensile strain has a different effect on these transport properties, and the improvement of the thermoelectric performance of InSe monolayer necessitates a complicated balance between these parameters *S*, *σ*, and *κ*. Figure 6 displays the calculated figure of merit with different strain as a function of chemical potential at 300 K, and it is obvious that the variation of ZT value under different strains strongly depends on the chemical potential and ZT maximum value can be effectively enhanced with the increase of strain. Without strain, the InSe monolayer has a peak ZT value of 0.36 at room temperature, which is close to that of silicene (0.36), germanene (0.41), and single-layer MoS_2_ (0.58) [53, 54], and lower than that of 2D monochalcogenides (1.29~2.63 at 700 K) [55]. Considering the high carrier mobility and superior mechanical flexibility, strained InSe monolayer is also a promising potential material for thermoelectric application. When tensile strain is applied, the weakened interatomic bond induces stronger anharmonicity. The increased phonon scattering rate, decreased phonon group velocity and phonon heat capacity together resulted in reduced lattice thermal conductivity, leading to an enhanced figure of merit. Previous theoretical calculations demonstrated that InSe monolayer can sustain a tensile strain over 20%, which is much larger than our predicted strains [20]. In the experiment, applying a strain on 2D materials are mostly through their interaction with substrates, which can be induced from heating [56], the lattice mismatch between epitaxial thin films [57], or bending of the 2D material on substrate [58, 59]. Actually, it is experimentally more common to apply uniaxial strain instead of biaxial strain. Based on the previous reports [20], a uniaxial strain may exhibit similar improvement on the thermoelectric properties of monolayer InSe.

## Conclusion

In conclusion, we systematically investigate the possible impact of biaxial tensile strain on the electronic, thermoelectric, and phonon transport properties for InSe monolayer by first-principles calculations. The bandgap decreases as the increase of tensile strain, leading to the reduced Seebeck coefficient. The tensile strain also induced stronger anharmonic scattering, and the reduction of lattice thermal conductivity could be attributed to the resulting increased phonon scattering rate, decreased phonon group velocity, and phonon heat capacity. The reduction of lattice thermal conductivity outweighs that of the Seebeck coefficient, thus bringing about an enhanced performance with the increase of tensile strain.

## Additional Files

Additional file 1: **Table S2.** Calculated bandgaps of InSe monolayer under different tensile strain. For sake of comparison, some previous theoretical and experimental (optical bandgap) results without strain are also listed. **Figure S1.** (a) The 2D elastic constant is obtained by parabola fitting total energy-strain relationship along *x* and *y* directions of monolayer InSe. (b) The band edge positions of conduction band and valence band with respect to the applied strain along *x* and *y* directions. Dotted line represents the linear fit, which defines deformation potential constant (DOCX 66 kb)

## Data Availability

The datasets generated and/or analyzed during the current study are available from the corresponding author on request.

## Abbreviation

2D: Two dimensional
CBM: Conduction band minimum
*τ*: Relaxation time
*C*_ph_: Phonon heat capacity
FET: Field-effect transistor
LA: Longitudinal acoustic phonon dispersion
PF: Power factor
*S*: Seebeck coefficient
TA: Transverse acoustic phonon dispersion
VBM: Valence band maximum
ZA: *z*-axis acoustic phonon dispersion
ZT: Figure of merit
*ε*_f_: Fermi level
*Κ*_e_: The thermal conductivity with the contributions from electronic carriers
*Κ*_l_: The thermal conductivity with the contributions from lattice
*σ*: Electrical conductivity
