# Supplementary material for: Strain Effect on Thermoelectric Performance of InSe Monolayer
Source: Nanoscale Res Lett. 2019 Aug 19;14:287. doi: 10.1186/s11671-019-3113-9 (PMC6702491; doi:10.1186/s11671-019-3113-9)
Supplement: Supplementary file 1 — Table S2. Calculated bandgaps of InSe monolayer under different tensile strain. For sake of comparison, some previous theoretical and experimental (optical bandgap) results without strain are also listed. Figure S1. (a) The 2D elastic constant is obtained by parabola fitting total energy-strain relationship along x and y directions of monolayer InSe. (b) The band edge positions of conduction band and valence band with respect to the applied strain along x and y directions. Dotted line represents the linear fit, which defines deformation potential constant (DOCX 66 kb) [file 11671_2019_3113_MOESM1_ESM.docx]

**Supporting Information**

**Strain effect on thermoelectric performance of InSe monolayer**

Qian Wang^1^, Lihong Han^1^, Liyuan Wu^1^, Tao Zhang^2^, Shanjun Li^2,*^, and Pengfei Lu^1,*^

*^1^**State Key Laboratory of Information Photonics and Optical Communications, Beijing University of Posts and Telecommunications, Beijing 100876, China.*

*^2^College of Electrical Engineering and Information Technology, Sichuan University, Chengdu 610065, China*

*Correspondence: lishanjun@scu.edu.cn; [photon.bupt@gmail.com](mailto:photon.bupt@gmail.com)

Table S1 The effective mass at the extrema of the valleys in the gap.

| **Electron effective mass (*m*_0_)** | | | | **Hole effective mass (*m*_0_)** | |
| --- | --- | --- | --- | --- | --- |
| G-M | M | K | G-K | G-M | G-K |
| 0.17 | 0.66 | 0.56 | 0.17 | 1.90 | 2.00 |

Table S2 Calculated band gaps of InSe monolayer under different tensile strain. For sake of comparison, some previous theoretical and experimental (optical band gap) results without strain are also listed.

|  | 0% | 2% | 4% | 6% | GGA | Exp. |
| --- | --- | --- | --- | --- | --- | --- |
| Gap (eV) | 1.67 | 1.43 | 1.21 | 1.01 | 1.41~1.55 [1-4] | 2.1 [5] |


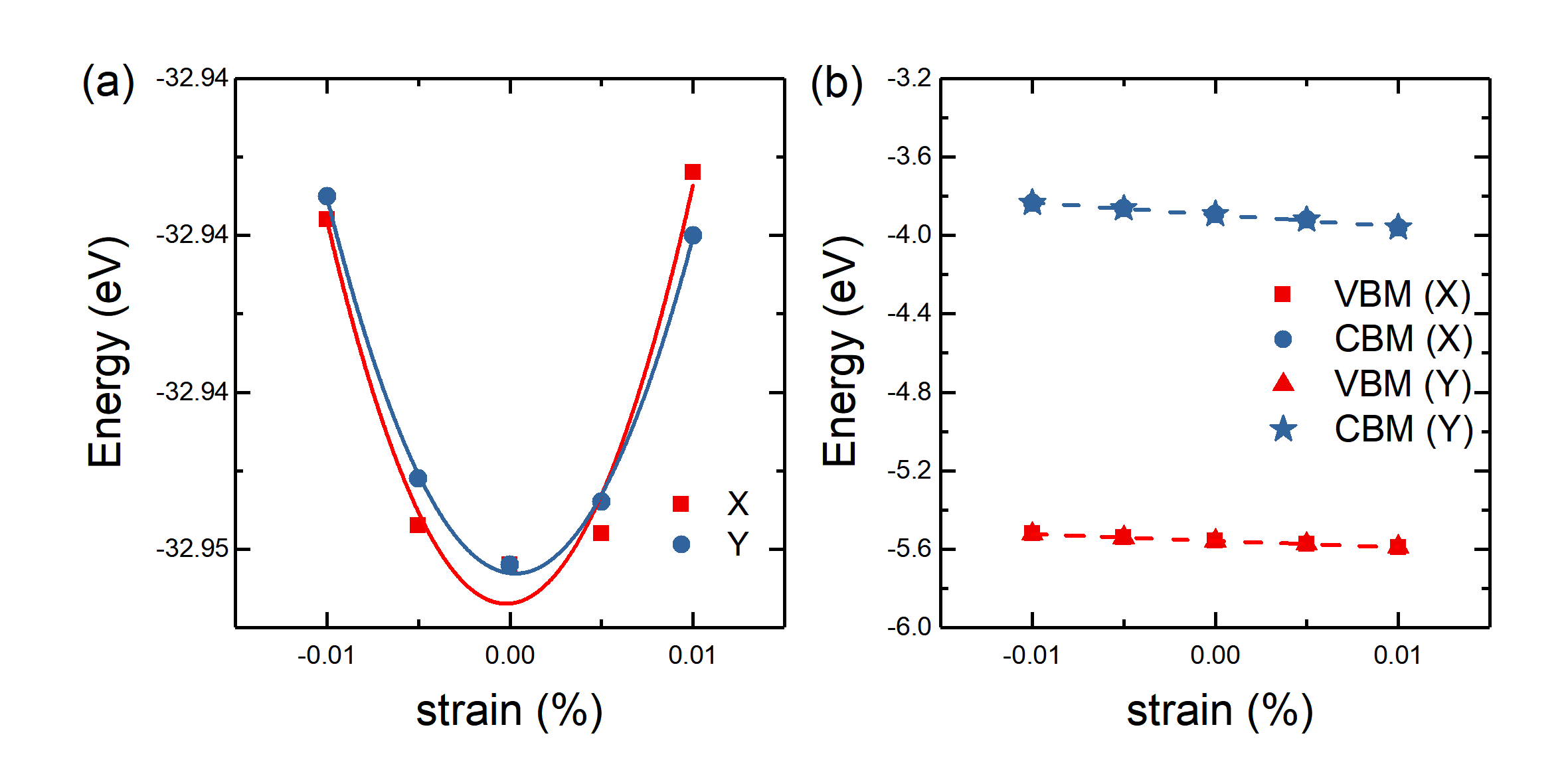


Fig. S1 (a) The 2D elastic constant is obtained by parabola fitting total energy-strain relationship along *x* and *y* directions of monolayer InSe. (b) The band edge positions of conduction band and valence band with respect to the applied strain along *x* and *y* directions. Dotted line represent the linear fit, which defines deformation potential constant.

**Reference**

[1] Guo, Y. and J. Robertson, *Band structure, band offsets, substitutional doping, and Schottky barriers of bulk and monolayer InSe.* Physical Review Materials, 2017. **1**(4): p. 044004.

[2] Ding, Y.-m., et al., *Enhancement of hole mobility in InSe monolayer via an InSe and black phosphorus heterostructure.* Nanoscale, 2017. **9**(38): p. 14682-14689.

[3] Hu, T., J. Zhou, and J. Dong, *Strain induced new phase and indirect–direct band gap transition of monolayer InSe.* Physical Chemistry Chemical Physics, 2017. **19**(32): p. 21722-21728.

[4 ] Hung, N.T., A.R. Nugraha, and R. Saito, *Two-dimensional InSe as a potential thermoelectric material.* Applied Physics Letters, 2017. **111**(9): p. 092107.

[5] Brotons-Gisbert, M., et al., *Nanotexturing to enhance photoluminescent response of atomically thin indium selenide with highly tunable band gap.* Nano letters, 2016. **16**(5): p. 3221-3229.
